# Lasp-1 Regulates Podosome Function

**DOI:** 10.1371/journal.pone.0035340

**Published:** 2012-04-13

**Authors:** Miriam Stölting, Christiane Wiesner, Vanessa van Vliet, Elke Butt, Hermann Pavenstädt, Stefan Linder, Joachim Kremerskothen

**Affiliations:** 1 Medizinische Klinik D, Abteilung für Molekulare Nephrologie, Universitätsklinikum Münster, Münster, Germany; 2 Institut für Medizinische Mikrobiologie, Virologie und Hygiene, Universitätsklinikum Eppendorf, Hamburg, Germany; 3 Institut für Klinische Biochemie, Universität Würzburg, Würzburg, Germany; University of Muenster, Germany

## Abstract

Eukaryotic cells form a variety of adhesive structures to connect with their environment and to regulate cell motility. In contrast to classical focal adhesions, podosomes, highly dynamic structures of different cell types, are actively engaged in matrix remodelling and degradation. Podosomes are composed of an actin-rich core region surrounded by a ring-like structure containing signalling molecules, motor proteins as well as cytoskeleton-associated proteins.

Lasp-1 is a ubiquitously expressed, actin-binding protein that is known to regulate cytoskeleton architecture and cell migration. This multidomain protein is predominantely present at focal adhesions, however, a second pool of Lasp-1 molecules is also found at lamellipodia and vesicle-like microdomains in the cytosol.

In this report, we show that Lasp-1 is a novel component and regulator of podosomes. Immunofluorescence studies reveal a localization of Lasp-1 in the podosome ring structure, where it colocalizes with zyxin and vinculin. Life cell imaging experiments demonstrate that Lasp-1 is recruited in early steps of podosome assembly. A siRNA-mediated Lasp-1 knockdown in human macrophages affects podosome dynamics as well as their matrix degradation capacity. In summary, our data indicate that Lasp-1 is a novel component of podosomes and is involved in the regulation of podosomal function.

## Introduction

Podosomes are highly dynamic adhesion structures that are constitutively formed in monocytic cells such as macrophages, dendritic cells, or osteoclasts, [1], [2], [3]. In addition, endothelial cells, smooth muscle cells, as well as glomerular podocytes, have been shown to form podosomes upon stimulation with cytokines, growth factors or phorbol esters [4], [5], [6], [7], [8]. Podosomes play a role in cell adhesion and matrix degradation, but their precise function in tissue invasion is still under consideration [9], [10]. On the molecular level, podosomes consist of an actin-rich core and associated proteins embedded in a ring-like structure of plaque molecules and signalling proteins.

Recent studies have shown that the formation of podosomes occurs very rapidly and starts with a clustering of podosome initiation factors. Subsequently in these cell type a recruitment of the actin polymerization machinery and a later maturation of podosomes occur [11], [12], [13].

A variety of molecules have been detected at podosomes including focal adhesion proteins (paxillin, vinculin, zyxin), actin assembly factors (Arp2/3, cortactin, palladin, Wiskott-Aldrich syndrome proteins ((N-)WASP), GTPases (arf6, cdc42, dynamin 2, rab5, rab7), transmembrane receptors, signalling molecules (src, tks5) and various matrix metalloproteinases [1], [2], [9], [10]. Most of these proteins can be clearly localized to either the podosome core or the ring structure.

LIM-and-SH3-domain-protein-1 (Lasp-1) is a multidomain protein that is known to regulate cytoskeleton dynamics [14], [15]. An increased level of Lasp-1 protein in carcinoma cells supports their motility and invasive character, whereas a reduced Lasp-1 expression inhibits chemotactic-induced migration of cultivated kidney cells [16], [17]. Lasp-1 is mainly localized at focal adhesions and F-actin-rich structures such as stress fibres and lamellipodia [18]. In addition, Lasp-1 shows a perinuclear accumulation at highly dynamic, vesicle-like microdomains [17], [18], [19]. A targeted disruption of the Lasp-1 gene in *Drosophila* causes a mislocation of cells in the testis [20], whereas Lasp-1 knockout mice display a disturbed gastric secretion [21].

The Lasp-1 protein is composed of an aminoterminal LIM domain, followed by two F-actin-binding nebulin (NEBU) domains, a Linker region and a carboxyterminal SH3 domain [15], [22]. The binding to F-actin is mainly mediated by the nebulin repeats of Lasp-1 [23]. The Lasp-1 SH3 domain interacts with zyxin, a mechanosensitive regulator of actin assembly, and controls its recruitment to focal adhesions [24], [25]. A second identified SH3-binding partner of Lasp-1 is palladin, a protein which is involved in actin assembly, too [8], [26].

Lasp-1 displays several phosphorylation motifs for cAMP-dependent serine/threonine kinases (PKA/PKC) as well as a substrate-recognizing sequence for the Abelson tyrosine kinase (Abl) [14], [27]. Furthermore, the subcellular distribution and physiological activity of Lasp-1 is controlled by phosphorylation at several sites [14], [28]. For example, induced Lasp-1 phosphorylation in fibroblasts prevents its localization at focal contacts and promotes its perinuclear enrichment [17].

The data presented in this study demonstrate that Lasp-1 is a component of podosomes in primary human macrophages and activated rat smooth muscle (A7r5) cells. Live cell imaging analysis, in combination with a siRNA-mediated knockdown approach demonstrates that Lasp-1 influences several parameters of podosome dynamics and also regulates podosome function by influencing their matrix degradation capacity.

## Materials and Methods

### Antibodies

Monoclonal antibodies directed against green fluorescent protein (GFP) were purchased from Clontech (Heidelberg, Germany); against zyxin, gelsolin, and paxillin from BD Biosciences (Heidelberg, Germany), against vinculin from Synaptic Systems (Göttingen, Germany) and Sigma-Aldrich (Hamburg, Germany), and against Arp2 from Abcam (Cambridge, UK). Polyclonal antibodies against Lasp-1 have been described earlier [28]. Fluorochrome-conjugated secondary antibodies, as well as phalloidin, coupled to the fluorescent dyes Alexa 488/−594/−568/−647 were purchased from Molecular Probes (Göttingen, Germany) and Invitrogen. Horseradish peroxidase-conjugated secondary antibodies were purchased from Dianova (Hamburg, Germany).

### Plasmids

The pcDNA3.1-Lasp-1 plasmid was described earlier [27]. Other Lasp-1 constructs, including truncation mutants, were either subcloned, or produced by PCR and cloning into the pEGFP-C1 or pDsRed1-N1 vectors (Clontech). The pDsRed2-C1 construct containing mouse cortactin cDNA (kind gift from A. Mak, Queen's University, Kingston, Canada) was described previously [13]. The pmRFP-Actin construct was a kind gift of M. Bähler, (University Münster, Münster, Germany). The EYFP-vinculin construct was a kind gift from A. Bershadsky (Weizmann Institute of Science, Rehovot, Israel), as well as MT1-MMP-mCherry from P. Chavrier (Institut Curie, Paris, France). pLifeact-TagGFP2 was purchased from ibidi (Martinsried, Germany).

### Cell culture, drug treatment and transfection

Human peripheral blood leukocytes were isolated by centrifugation of heparinized blood or buffy coats (kindly provided from F. Bentzien, University Medical Center Hamburg-Eppendorf, Germany) in Ficoll (Biochrom, Berlin, Germany). Monocytic cells were isolated with magnetic anti-CD14 antibody beads and MS+ separation columns (Miltenyi Biotec, Auburn, CA), according to the manufacturer's instructions and seeded onto glass coverslips at a density of 5×10^4^ or 35 mm plastic wells at a density of 1×10^6^. Cells were cultured in RPMI containing 20% cell-free autologous serum at 37°C, 5% CO_2_ and 90% humidity and differentiated into macrophages through plate adherence over 6 days. Autologous cell-free serum, which was isolated from whole blood using Serum Separation tubes (S-Monovette® from Sarstedt, Nümbrecht, Germany), filtrated through Stericup® Filter Units (Millipore) and used without previous heat inactivation. Medium was changed every 3–4 days.

A7r5 rat smooth muscle cells (ATCC) were grown on glass coverslips in high glucose Dulbecco's Modified Eagle Medium (DMEM) without phenol red, containing 10% fetal calf serum and 1× penicillin/streptomycin. For the expression of EGFP, mRFP or DsRed fusion proteins, A7r5 cells were transfected with plasmid DNA using Fugene HD reagent (Roche, Mannheim, Germany) following the manufacturer's instructions. 48 hours after transfection, podosome formation was induced by PDBu treatment for the indicated times. Transfection of macrophages was described previously [29], [30].

### Lysate preparation

Cells were either lysed directly into Laemmli sodium dodecyl sulphate (SDS) sample buffer and boiled for 5–10 minutes or scraped into lysis buffer (LB, 20 mM Tris-HCl (pH 7.5), 25 mM NaCl, 50 mM NaF, 15 mM Na_4_P_2_O_7_, 1% TritonX-100, 1 mM EDTA, *Complete* Protease Inhibitor, Roche, Mannheim, Germany) and lysed by 10 passes through a 26-gauge needle. Cell lysates were centrifuged at 14.000×g at 4°C and supernatants were stored at −80°C until further use.

### Western blotting

Western blotting was performed using standard techniques [31]. Antibodies were diluted in blocking buffer containing 5% skim milk powder in PBS/Tween. After washing, the membranes were incubated with respective secondary antibodies coupled to horseradish peroxidase. Finally, the membranes were washed and developed using the Lumilight chemiluminiscence detection kit (Roche) and X-ray film developer.

### Lasp-1 siRNA knockdown

Control siRNA duplexes (5′-TTCTCCGAACGTGTCACGTTT-3′, 20 nM, Qiagen, Hilden, Germany) as well as validated stealth siRNA oligo A (5′-AAGGTGAACTGTCTGGATAAG-3′, 20 nM, Invitrogen) targeting rat Lasp-1 mRNA were transfected into A7r5 cells with the use of Lipofectamine 2000 reagent (Invitrogen) according to the manufacturer's recommendations. Preparation of A7r5 cell lysates and immunofluorescence microscopy of cells grown on coverslips, respectively, were carried out 72 h posttransfection to determine the efficiency of the siRNA-mediated Lasp-1 knockdown.

Podosome formation in A7r5 Lasp-1 knockdown cells was induced by PDBu treatment (1 µM for 30 min) 72 h posttransfection. Cells were fixed and stained with phalloidin-Alexa594 to visualize the actin core of podosomes. The number of podosomes per cell after siRNA treatment was determined and quantitatively analyzed using a student t-test. At least 35 cells per coverslip from three different experiments were counted.

Human macrophages were transfected in two steps (78 h and 96 h in total) with two different siRNA duplexes (Oligo A: -5′-AAGGTGAACTGTCTGGATAAG-3′ and Oligo C:-5′-GCATGCTTCCATTGCGAGA-3′, 1 µM each, ThermoScientific (Bonn, Germany)) targeting human Lasp-1 mRNA or control siRNA targeting firefly luciferase (5′-AGGTAGTGTAACCGCCTTGTT-3′) using a MicroPorator MP-100 (PeqLab, Erlangen, Germany) with the following specifications: pulse voltage 1000 V, pulse width 40 ms, pulse number 2.

Preparation of lysates from siRNA-transfected cells was carried out 78 h and 96 h posttransfection to determine the efficiency of the siRNA-mediated Lasp-1 knockdown using Western blotting analysis.

### Immunofluorescence analysis

A7r5 cells grown on coverslips were fixed in 4% paraformaldehyde in PBS. Human macrophages were fixed with 3.7% formaldehyde in PBS and permeabilized in ice-cold acetone and 0.5% Triton X-100, respectively. Endogenous proteins were detected by subsequent incubation of the cells with primary and fluorochrome-coupled secondary antibodies for one hour at room temperature. After washing, specimen were mounted in Vectastain mounting medium (Vector Laboratories, Burlingame, CA) or Mowiol (Calbiochem, Schwalbach, Germany) containing *p*-phenylendiamine (Sigma-Aldrich) as anti-fading reagent, and sealed with nail polish. Samples were analyzed on a Leica photomicroscope (Leica, Wetzlar, Germany) attached to a Spot 2 Slider digital camera (Meyer Instruments, Houston, TX). Confocal microscopy was performed on a confocal laser scanning microscope (Leica) or a LSM510 Meta microscope (Carl Zeiss, Jena, Germany). Substitution of primary antibodies by non-immune serum served as negative controls.

For analyzing the number and diameter of podosomes, primary human macrophages were seeded on glass coverslips in a density of 1×10^5^ cells. After 6 h, the cells were fixed, permeabilized and stained with Alexa 488-coupled phalloidin for highlighting podosome cores. The numbers of podosomes were evaluated using ImageJ and GraphPad Prism software. In total, 168 cells were used from three different donors. For the diameter of the podosome cores, in total 24 cells from three different donors were analysed using Volocity 3D Image Analysis Software (PerkinElmer, Rodgau, Germany) and evaluated with Excel and GraphPad Prism software.

For analysing the number of podosomes contacted by MT1-MMP containing vesicles, MT1-MMP-mCherry was overexpressed for 18 h in primary human macrophages, then fixed, permeabilized and stained with Alexa 488 coupled phalloidin. The fluorescence intensity of single podosomes was measured using ImageJ software and evaluated with Excel and GraphPad Prism software. In total, 12 cells from three different donors were analysed.

### Live cell imaging

24 h posttransfection, A7r5 cells were seeded onto 35 mm μ-dishes (ibidi, Martinsried, Germany) and intracellular dynamics of recombinant EGFP-, mRFP- or DsRed-tagged proteins were analyzed with the live cell imaging system Biostation IM (Nikon, Duesseldorf, Germany). Therefore, an internal sealed chamber with humidified 5% CO_2_ and 37°C was used. Pictures from selected cells were taken every 30 sec over time periods of 5–30 min.

Primary human macrophages were seeded onto 12 mm glas bottom dishes (Willco Wells BV, Amsterdam, Netherlands). The dynamics of single podosomes or the area of all podosomes, were analysed 18 h posttransfection of Lifeact-TagGFP2. Pictures from single cells were taken every 10 sec over time periods of 30 min. For evaluation, in total eleven cells,from three different donors, were analysed, using Volocity 3D Image Analysis Software and GraphPad Prism. Rework of movies was done using After Effects CS5 (Adobe, Neu-Isenburg, Germany).

### Matrix degradation assay

A matrix degradation assay was performed as described earlier [32]. In brief, gelatin (from swine, Roth, Karlsruhe, Germany) was fluorescently labeled with NHS-rhodamine (ThermoScientific, Rockford, IL). Coverslips were coated with labeled matrix solution, fixed in 0.5% glutaraldehyde and washed with 70% ethanol and medium. SiRNA-transfected human macrophages were seeded on coated coverslips with a density of 8×10^4^ cells and fixed after 6 and 24 h, respectively.

Quantification of matrix degradation was performed using ImageJ software. Values were determined by measurement of the fluorescence intensity of the matrix in a single cell mode. Values of cells transfected with control siRNA were set at 100%. For comparability, laser intensity was not changed between measurements. For each value, 3×30 cells were evaluated. When indicated, differences between mean values were analyzed using the Student's *t* test. P<0.05 was considered as statistically significant.

## Results

### Lasp-1 is a component of the podosomal ring

**Figure 1:**
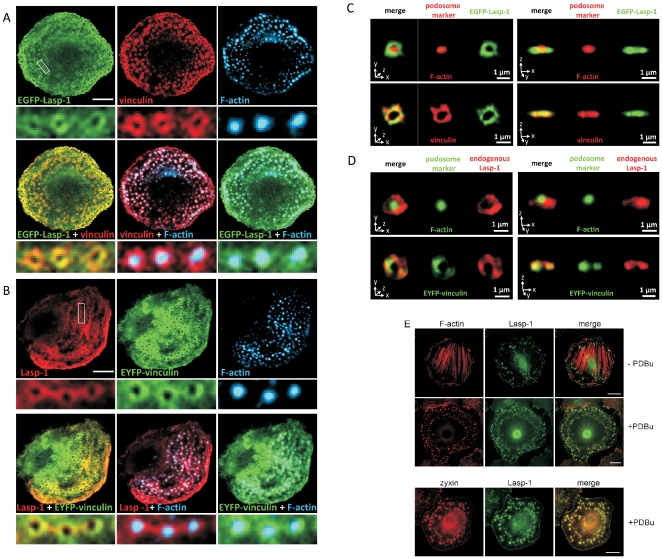
Lasp-1 is a component of the podosome ring structure. (**A, B**) Confocal micrographs of primary human macrophages transfected with constructs encoding (A) EGFP-Lasp-1 and (B) EYFP-Vinculin (green), respectively, and stained with vinculin- or Lasp-1-specific antibodies (with Alexa488- or Alexa 568-conjugated secondary antibody) for endogenous vinculin or Lasp-1, respectively, and Cy5-conjugated phalloidin for F-actin (blue). White box indicates detail images below. Bars represent 10 µm. (**C, D**) 3D reconstruction of single podosomes of primary human macrophages. Left panels: xyz mode, right panels: xzy mode for the same podosome. (C) Cells were transfected with EGFP-Lasp-1 and stained with Alexa568-phalloidin for F-actin (podosome core) or vinculin-specific antibody (with Alexa568-labeled secondary antibody) for vinculin (podosome ring structure; red). (D) Both untransfected cells (stained with Alexa488-phalloidin for F-actin; green) and cells transfected with EYFP-vinculin (green) were stained with Lasp-1-specific antibody (with Alexa568-labeled secondary antibody) for endogenous Lasp-1 (red). (**E**) Confocal micrographs of a rat smooth muscle cell (A7r5), stained with Alexa-594 phalloidin for F-actin (red, upper panel) or zyxin-specific antibody (with Alexa594-labeled secondary antibody) for endogenous zyxin (red, lower panel) and co-stained with Lasp-1-specific antibody (with Alexa488-labeled secondary antibody) for endogenous Lasp-1 (green). Lasp-1 and its binding partner zyxin colocalize at F-actin-rich podosomes (merge) in PDBu-treated A7r5 cells. Bars represent 25 µm.

To analyze a putative podosomal localization of Lasp-1, we used indirect immunofluorescence and confocal microscopy (Fig. 1A–E). In transfected human macrophages, Lasp-1 tagged with the enhanced green fluorescent protein (EGFP-Lasp-1) colocalized with endogenous vinculin at the ring structure of podosomes surrounding the actin core (Fig. 1A). Similar results were obtained with macrophages encoding vinculin-tagged enhanced yellow fluorescent protein (EYFP) and stained for endogenous Lasp-1 and F-actin (Fig. 1B). A more detailed analysis of single podosomes from macrophages using the Volocity software package confirmed that both endogenous as well as overexpressed Lasp-1 localize to the podosomal ring structure, where they partially colocalize with vinculin (Fig. S1A/B).

Whereas macrophages generate podosomes constitutively, in A7r5 smooth muscle cells, podosome formation can be induced by stimulation of protein kinase C with the phorbol ester PDBu. Using this sytem, we observed that in unstimulated A7r5 cells, Lasp-1 was mainly found at focal adhesions that anchors stress fibres to the basal membrane (Fig. 1E). Concomitant with the PDBu-induced cytoskeletal rearrangement, Lasp-1 was enriched at podosomes in PDBu-treated A7r5 cells, where it colocalized with the podosome component zyxin (Fig. 1E).

### Podosomal localization of Lasp-1 truncation mutants

**Figure 2:**
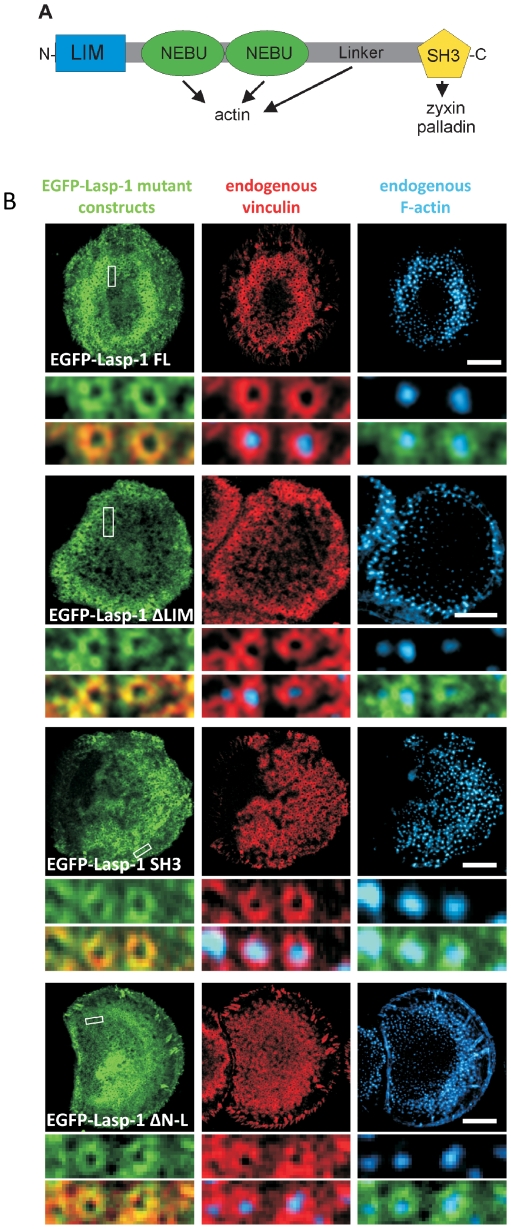
Lasp-1 mutants lacking either the LIM domain, the NEBU repeats and the Linker region, or the SH3 domain are still localized at podosomes. (**A**) Domain structure of Lasp-1 comprising an aminoterminal LIM domain, two nebulin (NEBU) repeats and an adjacent Linker region mediating actin binding, and a carboxyterminal SH3 (Src homology 3) domain that binds zyxin and palladin (N = aminoterminus, C = carboxyterminus). (**B**) Confocal micrographs of primary human macrophages transfected with EGFP-Lasp-1 deletion constructs (green) and stained with vinculin-specific antibody (with Alexa568-conjugated secondary antibody) for vinculin (red) and Cy5- phalloidin for F-actin. Constructs include EGFP-Lasp-1 wildtype (wt), EGFP-Lasp-1ΔLIM (lacking the LIM domain), EGFP-Lasp-1ΔSH3 (lacking the SH3 domain), and EGFP-Lasp-1ΔN-L (lacking the NEBU repeats and the Linker region). White boxes indicate detail images below. Bars represent 10 µm.

Lasp-1 is a multidomain protein that can bind different proteins including F-actin as well as the known podosomal components zyxin and palladin (Fig. 2A). To determine the specific Lasp-1 domain(s) that mediate(s) its podosomal recruitment, we analyzed the distribution of various EGFP-tagged Lasp-1 truncation mutants in transfected macrophages. EGFP fusion proteins encoding only isolated Lasp-1 domains (SH3, LIM, NEBU) displayed a diffuse cytosolic distribution indicating that a single Lasp-1 domain is not sufficient to establish a podosomal localization (data not shown). In contrast, EGFP-Lasp-1 truncation mutants that lack either the aminoterminal LIM domain, the internal NEBU repeats and the adjacent linker region, or the carboxyterminal SH3 domain all localized to podosomes of transfected macrophages (Fig. 2B), and also to vinculin-positive focal adhesions in A7r5 cells (Fig. S2A/B). These data demonstrate that a combination of at least two distinct functional domains is involved in the recruitment of Lasp-1 to podosomes.

### Lasp-1 is involved in early stages of podosome biogenesis

**Figure 3:**
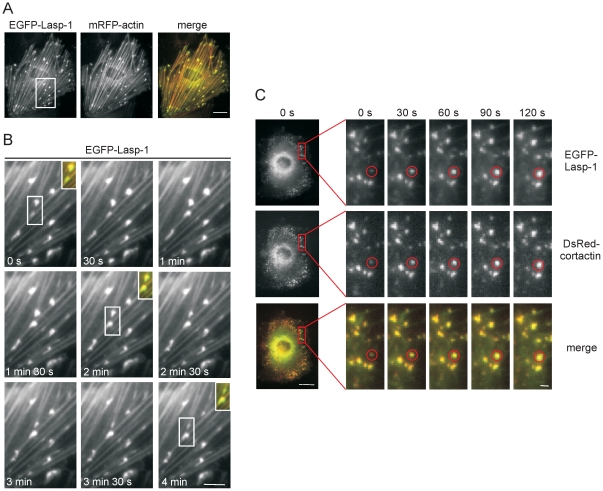
Lasp-1 recruitment is associated with early stages of podosome biogenesis. (**A**) Colocalization of recombinant EGFP-Lasp-1 and recombinant mRFP-actin at podosomes in PDBu-treated A7r5 cells. The white box marks the enlargement shown in Fig. 3B. Bar represents 25 µm. (**B**) Appearance of EGFP-Lasp-1 during assembly and disassembly of podosomes (white boxes) in PDBu-treated cells over a period of 4 min. Inserts in the pictures taken at 0 s, 2 min. and 4 min. show the merged EGFP-Lasp-1 (green) and mRFP-actin (red) signals in two individual podosomes. Bar represents 2 µm. (**C**) Colocalization of EGFP-Lasp-1 (upper row) and DsRed-cortactin (middle row, merged pictures in the lower row) during initial stages of podosome assembly in PDBu-treated A7r5 cells over a period of 2 min. An individual podosome is marked by a red circle. Bars represent 25 µm (left panel) or 2 µm (right panels), respectively.

Next, live cell imaging experiments with A7r5 cells were performed to study Lasp-1 dynamics during podosome biogenesis (Fig. 3). EGFP-Lasp-1 colocalizes with recombinant mRFP-actin at podosomes of PDBu-treated A7r5 cells (Fig. 3A). Time-lapse analysis revealed that EGFP-Lasp-1 is recruited to podosomes during their assembly and remains there until disassembly after 3–4 minutes (Fig. 3B).

The podosomal component cortactin is known to be crucial for early steps in podosome biogenesis as the clustering of initiation factors occurs [13]. When we compared DsRed-cortactin and EGFP-Lasp-1 in double-transfected, PDBu-treated A7r5 cells, both recombinant proteins displayed comparable dynamics during the rapid assembly process (Fig. 3C).

### Lasp-1 knockdown, podosome formation and matrix degradation

To investigate the functional relevance of Lasp-1 for podosome formation, we used a siRNA-based approach to knock down Lasp-1 in PDBu-treated A7r5 smooth muscle cells. Compared to cells transfected with control siRNA, there was a notable reduction of the protein level of Lasp-1 in cells transfected with specific siRNA against Lasp-1 (Fig. S3A/B). Interestingly, podosomes were still apparent after Lasp-1 knockdown in PDBu-treated cells (Fig. S3B), indicating that Lasp-1 is not an essential factor for podosome assembly in A7r5 cells.

Next, we transferred the siRNA approach to human macrophages. Transfection of cells with two different siRNAs that target human Lasp-1 (Oligo A or C) resulted in a marked decrease of Lasp-1 expression (Fig. S4A). However, immunofluorescence analysis revealed that similar to the results obtained with stimulated A7r5 cells, Lasp-1 siRNA transfected macrophages were still able to form podosomes with a core region that contains F-actin and the marker proteins Arp2 and gelsolin (Fig. S4B) as well as a podosome ring structure that contains vinculin and paxillin (Fig. S4C). These data suggest that the main structure of podosomes is not altered in Lasp-1-deficient macrophages.

**Figure 4:**
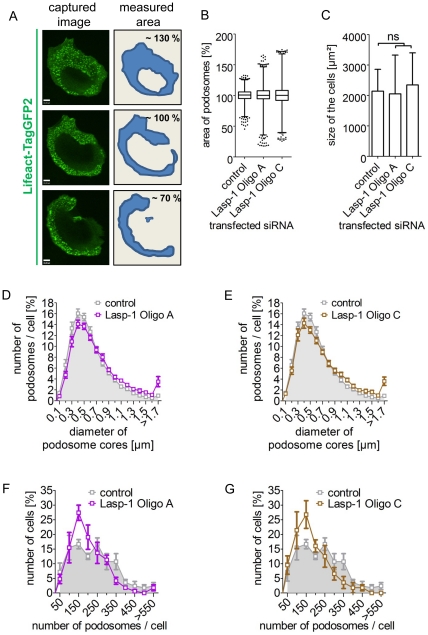
Impact of a siRNA-mediated knockdown of Lasp-1 on podosome numbers and structure in macrophages. Primary human macrophages were treated with control siRNA or Lasp-1 specific siRNA (Oligo A or C). (**A–C**) Podosome-covered area was analyzed by life cell imaging, with example shown in (A). Pictures were taken every 30 sec. over 30 min. (n = 3×4 cells). Average area per cell was set at 100%. Values were plotted as box graphs, with whiskers from 1–99% (B). Note higher variability of podosome-covered areas in cells treated with Lasp-1-specific siRNA. (C) Measured cell size from cells used in (B). (means+SD; ns = not significant). (**D–G**) Diameter of the podosome cores (D, E) and number of podosomes per cell (F, G) were evaluated from cells treated with Lasp-1-specific siRNA (Oligo A (D, F; purple); Oligo C (E,G; brown)) or control siRNA (grey). Insets (d, e) show details taken from graph (means ±SE, n = 3×8 (D, E); means±SE n = 3×56 (F, G)).

Next, we measured whether a Lasp-1 knockdown in macrophages affects podosome size and number (Fig. 4). Cells that were treated with specific siRNA against Lasp-1 showed slightly more podosomes with a larger diameter of the core structure (Fig. 4 D/E). However, the number of podosomes per cell was decreased compared to cells treated with control siRNA (Fig. 4 F/G). Furthermore, overall dynamic of the podosome field per cell was quantified by measuring the total podosome-covered area per cell over time (Fig. 4 A–C; Video S1). Macrophages transfected with Lasp-1-specific siRNA (Oligo A or C) showed a higher variability of this value, compared to control cells, indicating increased overall podosome dynamics. By contrast, the size of these cells showed no difference to controls (Fig. 4 B/C)

**Figure 5:**
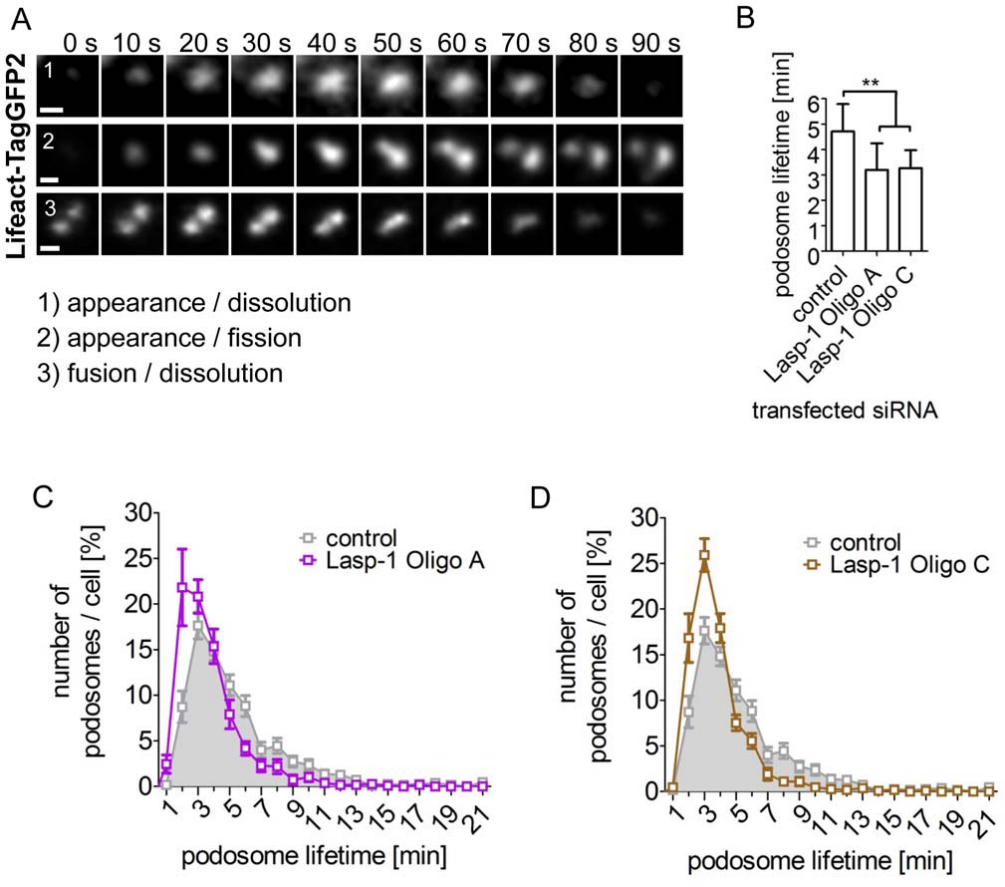
Podosome lifetime is altered in Lasp-1 knockdown macrophages. Primary human macrophages were treated with Lasp-1-specific siRNA (Oligo A or C) or control siRNA. (**A**) Examples for podosome dynamics included in the analysis. Shown are single podosomes of cells co-transfected with siRNA and Lifeact-TagGFP2. Podosome lifetime is defined as 1) appearance to dissolution, 2) appearance to fission, or 3) fusion to dissolution. (**B–D**) Measurement of podosome lifetime from cells treated with Lasp-1-specific siRNA, compared to controls. Double asterisks indicate values highly significant different from controls with *P*<0.004 (means+SD, n = 3×4) (B). (C, D) Graph showing detailed podosome lifetime analysis, data from (B), of cells transfected with Lasp-1-specific siRNA (Oligo A (C; purple); Oligo C (D; brown), compared to controls (C, D; grey; (means+SE; n = 3×4;)).

To determine the role of Lasp-1 in the dynamics of single podosomes, we analyzed podosome lifetime, which includes 1) appearance to dissolution, 2) appearance to fission, and 3) fusion to dissolution of single podosomes (Fig. 5 A). Importantly, macrophages treated with Lasp-1 specific siRNA (Oligo A or C; Figure 5B) showed a significant decrease (approx. 30%) of mean podosome lifetime, and especially the number of podosomes showing short lifetimes (1–3 min) was significantly increased. (Fig. 5 C/D).

**Figure 6:**
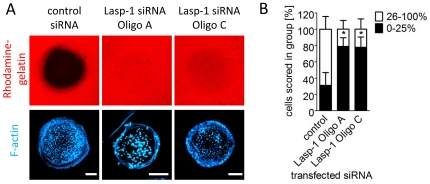
Knockdown of Lasp-1 impairs the matrix degradation capacity of podosomes from human macrophages. **A**) Confocal micrographs of macrophages transfected with control siRNA or siRNAs (Oligo A or C) specific for human Lasp-1, and seeded on rhodamine-labeled gelatin (red). Cells were fixed after 6 h and stained with Cy5-phalloidin for F-actin (blue). Matrix degradation is visible as dark areas by concomitant loss of the fluorescent label. White bars represent 10 µm. (**B**) Statistical quantification of matrix degradation in cells treated with control siRNA or siRNAs specific for Lasp-1. Measurement of the fluorescence intensity of the degraded area was used to evaluate the degree of degradation. Complete absence of signal was set as 100% degradation and the evaluated cells were scored into groups depending on the degree of matrix degradation (0%–25%; 26%–100%). Single asterisks indicate values significantly different from control with a *P* value<0.05 (means with n = 3×30).

As the degradation of extracellular matrix (ECM) is one of the main functions of podosomes [33], [34], we analyzed the putative effect of a Lasp-1 knockdown on the matrix degradation capacity. For this assay, we used macrophages pre-treated with control siRNA or siRNA targeting Lasp-1 (Oligo A or C), seeded on coverslips coated with fluorescent-labeled gelatin and analyzed after 6 hrs of degradation. After this period, a significant reduction of degradation could be observed in cells treated with siRNA against Lasp-1 (Fig. 6A). This could also be quantified by measuring the fluorescence intensity of the degraded area and resulted in a significant difference compared the degradation level of control cells (Fig. 6B).

Matrix degradation at podosomes requires the recruitment of matrix metalloproteinases including MT1-MMP [34]. To test whether Lasp-1 is involved in this recruitment process, we analyzed the amount of MT1-MMP-mCherry-containing vesicles at podosomes from control or Lasp-1 siRNA-treated macrophages. No effect of a Lasp-1 knockdown on the podosomal localization of MT1-MMP was detected (Fig. S5A/B).

## Discussion

Podosomes are highly dynamic (lifetime ca. 2–12 min), actin-rich structures at the substrate attached site of cells [1], [9]. To date, various cell types are known that form podosomes constitutively (e.g. osteoclasts, macrophages; [1], [2], [10], [33]) or upon stimulation (e. g. A7r5 smooth muscle cells; [4], [6], [29], [34], [35], [36]).

The actin-binding protein Lasp-1 is known to localize at stable actin-rich structures like focal adhesions and stress fibres, but can also be found at highly dynamic dorsal membrane ruffles [15], [17]. These findings, together with the already known interaction between Lasp-1 and zyxin and palladin, led us to investigate whether Lasp-1 is a component of podosomes, too.

In the current study, we observed Lasp-1 localization at podosomes in smooth muscle cells and human macrophages, respectively (Fig. 1; Fig. S1). Our immunofluorescence analyses revealed, that Lasp-1 is localized in the ring structure of podosomes and displays a distribution that is similar to that of other adhesion plaque proteins such as vinculin, zyxin and paxillin [1], [12], [37]. Our data from experiments with Lasp-1 truncation mutants demonstrated that a proper podosomal localization requires the combinantion of at least two functional domains of the protein. Neither the NEBU repeats that are known to associate with F-actin, nor the SH3 domain that binds to paxillin and zyxin were sufficient or necessary to target a EGFP fusion protein to podosomes (Fig. 2). These findings are in line with a recent study demonstrating, that various truncation mutants of Lasp-1 lacking different domains are still recruited to focal adhesions [17].

We observed a comparable localization of Lasp-1 and the early podosome marker cortactin at sites of initial podosome formation (Fig. 3B). Cortactin is known to be crucial for the initiation of actin polymerization at pre-podosome structures [5], [12], [13], [38], [39]. As Lasp-1 and cortactin display similar dynamics during podosome biogenesis, we speculated that Lasp-1 is associated with early stages of podosome biogenesis, too. To prove this, we used a siRNA-based approach to knock down Lasp-1 in PDBu-treated A7r5 cells (Fig. S3). Interestingly, we observed no differences in the overall number of podosomes in Lasp-1 knockdown A7r5 cells. However, in human macrophages with a decreased Lasp-1 expression, we observed alterations in several parameters of podosomes: decreased lifetime, smaller diameter and decreased podosome numbers per cell. Moreover, also the degradation capacity of podosomes was diminished in these cells (Fig. 5/6). These data point to potential cell type-specific differences in the recruitment or regulation of both structural and functional podosome components. In this context, it should also be mentioned that macrophages form podosomes constitutively, whereas podosome formation in A7r5 smooth muscle cells is induced by stimulating PKC, and thus not directly comparable. In a similar scenario, knockdown of cortactin in carcinoma cells resulted in decreased matrix degradation ability of the podosome-related invadopodia [40]. In these cells, secretion of the matrix metalloproteinases (MMP) MMP-2 and MMP-9 as well as cell surface exposure of the transmembrane isoform MT1-MMP was found to closely correlate with cortactin expression levels. Although we did not detect a direct correlation between Lasp-1 expression and the recruitment of MT1-MMP-containing vesicles to podosomes, our results are consistent with the idea that Lasp-1 may be important for the local release of lytic enzymes at matrix degrading podosomes in macrophages.

In summary, our study shows that Lasp-1 is a novel component of the podosomal ring structure. Although Lasp-1 is recruited to podosomes at early stages of their assembly, the protein is probably not necessary for the initiation of podosome formation. However, Lasp-1 is involved in of the regulation of several podosome parameters including size, number and lifetime and also regulates the matrix degradation capacity of podosomes. These activities reveal Lasp-1 as a novel important regulator of podosomes and also point to Lasp-1 as a potential target for the modulation of invasive cell migration.

## Supporting Information

Figure S1 **Lasp-1 is a component of the podosome ring structure.** 3D reconstruction of single podosomes of primary human macrophages. Fluorescence micrographs (shown and described in Figure 1 C, D) were converted *in silico* into an isosurface mode using Volocity 3D Image Analysis Software. Left panels: xyz mode, right panels: xzy mode for the same podosome. It is important to note that common localization of proteins to either core or ring structures of podosomes does not necessarily imply exact colocalization, as apparently subdomains exist within both structures. Thus, both vinculin and Lasp-1 clearly localize to the podosome ring, but they only overlap partially.
(TIF)

Figure S2 **Recruitment of Lasp-1 truncation mutants to focal adhesions.** (**A**) A7r5 cells were transfected with constructs encoding EGFP-tagged Lasp-1 wildtype (wt) or various truncation mutants that lack individual domains. Fixed cells were stained for the focal adhesion marker protein vinculin using specific primary antibody (with Alexa594-labeled secondary antibody) (red). Scale bars represent 5 µM. (**B**) Lack of localization of individual Lasp-1 domains at vinculin-positive focal adhesions. A7r5 cells were transfected with constructs encoding EGFP-tagged Lasp-1 domains. Fixed cells were stained for the focal adhesion marker protein vinculin using specific primary antibody (with Alexa594-labeled secondary antibody) (red). Scale bars represent 5 µM.
(TIF)

Figure S3 **SiRNA-mediated Lasp-1 knockdown does not prevent podosome formation in PDBu-treated A7r5 cells.** (**A**) Western blot analysis of Lasp-1 and ß-tubulin expression in A7r5 cells transfected with control siRNA (lane 1) or siRNA targeting Lasp-1 mRNA (Oligo A, lane 2) for 72 h. (**B**) A7r5 cells, transfected with Lasp-1 specific siRNA (Oligo A) or control siRNA, respectively and treated with PDBu, were fixed and stained with Lasp-1-specific antibody (and Alexa488-conjugated secondary antibody) for endogenous Lasp-1 and Alexa 594-phalloidin for F-actin. Treatment of A7r5 cells with Lasp-1 siRNA (right column) decreases the detectable Lasp-1 protein level, but does not prevent PDBu-induced formation of F-actin-rich podosomes. Bar represents 25 µM.
(TIF)

Figure S4 **Knockdown of Lasp-1 in human macrophages does not affect the overall structure of podosomes.** (**A**) Western Blot analysis of Lasp-1 and ß-actin expression in human macrophages transfected twice with control siRNA (lanes 1 and 3) or two different oligonucleotides (lanes 2 (Oligo A) and 4 (Oligo C)) targeting Lasp-1 mRNA. (**B,C**) Confocal micrographs of primary human macrophages treated with control siRNA or Lasp-1-specific siRNA (Oligo A or C). Cells were fixed and stained with Alexa568-phalloidin for F-actin (highlighting podosome cores; red) and with Arp2- or gelsolin-specific antibodies (with Alexa488-conjugated secondary antibody) for Arp2 or gelsolin (podosome cores; green) or with vinculin- or paxillin-specific antibodies (with Alexa488-conjugated secondary antibody) for vinculin and paxillin (podosome ring structure; green), respectively. White bars indicate 10 µm.
(TIF)

Figure S5 **Lasp-1 expression does not correlate with a recruitment of MT1-MMP to podosomes in macrophages.** (**A**) Confocal micrograph of a macrophage transfected with MT1-MMP-mCherry (red), fixed and stained with Alexa488-phalloidin to stain F-actin (highlighting podosome cores; green). Inset shows detail image indicated by white box, with examples of a MT1-MMP-mCherry containing vesicle contacting a podosome (arrow) and a vesicle adjacent to, but not contacting a podosome (arrowhead). Bar indicates 10 µm. (**B**) Primary human macrophages were treated with Lasp-1-specific siRNA (Oligo A or C) or unspecific control siRNA and cotransfected with MT1-MMP-mCherry. Cells were fixed and stained with Alexa488-phalloidin. Percentages of podosomes per cell in contact with MT1-MMP-mCherry containing vesicles were.evaluated by measuring the fluorescence intensity. Values are means (+SE; n = 3×4; n.s. = not significant).
(TIFF)

Video S1 **Knockdown of Lasp-1 mediates higher variability of the area of podosomes in macrophages.** Confocal time lapse series of primary human macrophages pre-treated with control and Lasp-1-specific siRNA (Oligo A or C), respectively, expressing Lifeact-TagGFP2 (green) highlighting F-actin (exposure time at 488 nm: 359 ms, acquisition rate: 1 image/30 sec; frame rate: 10 f/s; sequence: 30 min). Note higher variability of the area of podosomes of cells treated with Lasp-1 specific siRNA compared to control cell.
(MOV)
