# Supplementary figures and images for: Lasp-1 Regulates Podosome Function
Source: PLoS One. 2012 Apr 13;7(4):e35340. doi: 10.1371/journal.pone.0035340 (PMC3325968; doi:10.1371/journal.pone.0035340)

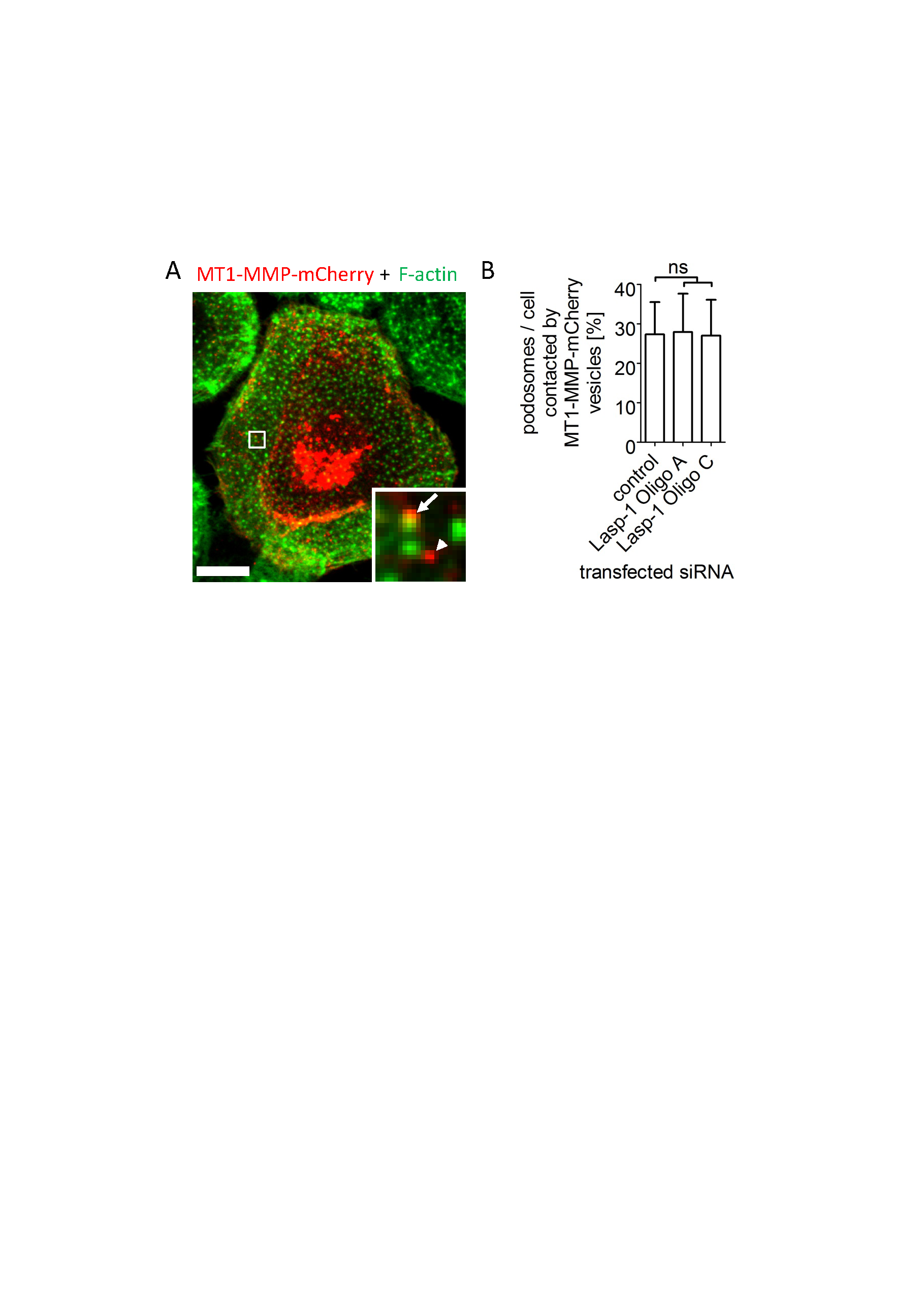

Supplement: Figure S5 — Lasp-1 expression does not correlate with a recruitment of MT1-MMP to podosomes in macrophages. (A) Confocal micrograph of a macrophage transfected with MT1-MMP-mCherry (red), fixed and stained with Alexa488-phalloidin to stain F-actin (highlighting podosome cores; green). Inset shows detail image indicated by white box, with examples of a MT1-MMP-mCherry containing vesicle contacting a podosome (arrow) and a vesicle adjacent to, but not contacting a podosome (arrowhead). Bar indicates 10 µm. (B) Primary human macrophages were treated with Lasp-1-specific siRNA (Oligo A or C) or unspecific control siRNA and cotransfected with MT1-MMP-mCherry. Cells were fixed and stained with Alexa488-phalloidin. Percentages of podosomes per cell in contact with MT1-MMP-mCherry containing vesicles were.evaluated by measuring the fluorescence intensity. Values are means (+SE; n = 3×4; n.s. = not significant). (TIFF) [file pone.0035340.s005.tif]
